# Genetic ancestry, skin color and social attainment: The four cities study

**DOI:** 10.1371/journal.pone.0237041

**Published:** 2020-08-19

**Authors:** Dede K. Teteh, Lenna Dawkins-Moultin, Stanley Hooker, Wenndy Hernandez, Carolina Bonilla, Dorothy Galloway, Victor LaGroon, Eunice Rebecca Santos, Mark Shriver, Charmaine D. M. Royal, Rick A. Kittles

**Affiliations:** 1 Division of Health Equities, Department of Population Sciences, City of Hope Medical Center, Duarte, California, United States of America; 2 Department of Health Disparities Research, M.D. Anderson Cancer Center, Houston, Texas, United States of America; 3 Department of Medicine, Section of Cardiology, University of Chicago, Chicago, Illinois, United States of America; 4 Departamento de Medicina Preventiva, Faculdade de Medicina, Universidade de São Paulo, Butanta, Brazil; 5 Utah Department of Health, Salt Lake City, Utah, United States of America; 6 Department of Anthropology, The Pennsylvania State University, University Park, Pennsylvania, United States of America; 7 Department of African & African American Studies, Duke University, Durham, North Carolina, United States of America; Universitat Pompeu Fabra, SPAIN

## Abstract

**Introduction:**

The Black population in the US is heterogeneous but is often treated as monolithic in research, with skin pigmentation being the primary indicator of racial classification. Objective: This paper examines the differences among Blacks by comparing genetic ancestry, skin color and social attainment of 259 residents across four US cities—Norman, Oklahoma; Cincinnati, Ohio; Harlem, New York; and Washington, District of Columbia.

**Methods:**

Participants were recruited between 2004 and 2006 at community-based forums. Cross-sectional data were analyzed using chi-square tests, correlation analyses and logistic regression.

**Results:**

There were variations in ancestry, melanin index and social attainment across some cities. Overall, men with darker skin color, and women with lighter skin color were significantly more likely to be married. Darker skin individuals with significantly more West African ancestry reported attainment of graduate degrees, and professional occupations than lighter skin individuals.

**Conclusions:**

Our findings suggest differences in skin pigmentation by geography and support regional variations in ancestry of US Blacks. Biomedical research should consider genetic ancestry and local historical/social context rather than relying solely on skin pigmentation as a proxy for race.

## Introduction

Race in the U.S. is largely based on skin color and ancestral history and continues to be an important variable in social science and biomedical research. The scholarship has largely examined the data across ethnic or racial classifications using gradations of skin pigmentation [1–3]. But skin color, while central to the discussion on race and genetics [4], is an imprecise proxy that has the potential to mask important information about social context and genetic ancestry [5].

Social context, which emerges at the intersection of historical, political, structural and personal forces, shapes the way people live and interpret their world [6]. Despite the centrality of social context in understanding actions and resulting outcomes, research on skin color has not given full treatment to issues related to culture and local histories, and Blacks (individuals of African descent including African American, African, and Caribbean persons) from different U.S. geographic regions are often treated as a homogenous group. The extant literature, for example, reports darker skin is associated with poorer overall health, and greater perceptions of discrimination and racism [2, 3, 7–9]. Skin color is understood as a determinant of social attainment as reflected in individuals’ occupation, income and marital status [10, 11]. However, these outcomes may vary by geographical regions in response to unique local experiences, historical events, and sociopolitical orientations.

An example of this variability is the socioeconomic differences between Blacks in northern and southern states, or between immigrant and U.S.-born Blacks. For reasons grounded in the legacy of slavery, Blacks who moved to the North in the Great Migration enjoyed greater economic gains than their counterparts who remained in the South [12]. Similarly, immigrant Blacks are more likely than U.S.-born Blacks to be college educated and tend to have higher median household income [13]. These differences in educational attainment and income drive differences in other social markers, such as health status, that may not directly correlate with skin color. Stratifying race data by region, therefore, may capture more closely the heterogeneity of the U.S. Black population and provide a more nuanced understanding of how skin color is associated with social attainment.

This paper examines the differences among U.S. Blacks by comparing residents across four cities in the United States. The four cities—Norman, Oklahoma (OK); Cincinnati, Ohio (OH); Harlem, New York (NY); and Washington, District of Columbia (DC)—have diverse historical experiences that shape their development and the profiles of modern-day Black residents. Prior to the 1800s, Norman, Oklahoma and Cincinnati, Ohio were primarily populated by White Americans [14, 15]. But, between 1826 and 1829, Cincinnati’s population of Blacks made up about 10% of the population [16]. This increased racial tensions and gave rise to laws and policies related to education and housing (i.e., Black code or redlining) that were intended to segregate Blacks and “keep them in their place”. During the same period, Oklahoma was also experiencing a demographic shift as thousands of Native Americans were forcibly relocated following the passage of the United States Indian Removal Act of 1830 [14, 15]. The Native Americans embarked on the arduous 1000-mile journey with their enslaved Blacks (known as *Freedmen*), dubbing the trek *The Trail of Tears* [14, 17] because of the immense suffering and loss of life. Conflict between Native Americans and the enslaved Black community ensued concerning the lineage of Blacks, some of whom had native American ancestry, and their entitlement to land from the Curtis Act of 1898 [14, 15, 18]. These historical tensions are manifested in the structure of today’s society as both Cincinnati and Norman remain racially divided and predominantly White communities.

On the contrary, DC and New York have emerged as cities with large Black populations. DC became a desirable location for many Black Americans, because it was recognized as a free state in 1862, prior to the emancipation proclamation. Howard University, a historically Black college and university, was founded in 1867 for Black scholars and continues to be the capstone of Black education and culture; and in 1957, DC residents elected the first Black Mayor [19]. To date, DC has retained the largest percentage of Black residents of any city in the United States, being comprised of more than 50 percent Blacks. Similarly, for NY, the mass migration of Blacks to Harlem shifted the demographics of the city from 33% to 70% Blacks in the 1920s and 1930s and gave rise to a cultural and artistic revolution that became known as the Harlem Renaissance. The great depression, however, sparked the decline of the economy and the population of Blacks in Harlem, with many migrating to neighboring cities in search of improved conditions and opportunities [19]. Many of these historical patterns were localized and created different population histories for Africans in America. Here we show the variability in four AA communities with differing local histories.

In addition to considering geographical and historical experiences in studies on skin color, incorporating information on genetic ancestry into this discourse will provide explanations for biological variations that are not fully explained by cultural and environmental factors [4]. Genetic ancestry provides a biological framework for explaining some of the differences that can be identified within groups and allows investigators to tease apart genetic and non-genetic effects and risk factors for complex disease. In investigations of the genetic influence of complex diseases in AAs complex diseases there may be confounding factors that influenced the association between genetic ancestry and disease phenotypes that many times are not included in the statistical modeling. Nonetheless, genetic ancestry captures the heterogeneity in ancestral genomic contributions in African American populations, and the use of genetic ancestry allows one to investigate the relative effect of ancestry-related genetic factors on disease phenotypes, before investigators even attempt to identify specific genomic regions or polymorphisms that are associated with diseases that disproportionately impact racial/ethnic groups. The interaction between genes and environment is responsible for the majority of variations in human phenotypes [20]. However, differences in genetic variability are not precisely correlated with self-identified race/ethnicity (SIRE) [21].

This paper highlights biological, social, and economic within group differences in the United States Black population by comparing skin color, genetic ancestry, and social attainment across four cities. The purpose of this study is to detail the heterogeneity of the U.S. Black population and highlight the danger in treating the group as monolithic for research convenience.

## Materials and methods

### Recruitment and data collection

Participants were recruited between 2004 and 2006 at community-based forums in Norman, Oklahoma (OK); Cincinnati, Ohio (OH); Harlem, New York (NY); and Washington, District of Columbia (DC). The forums allowed attendees to participate in genetic ancestry testing after information on testing was provided and organizers responded to questions from community members. We collaborated with local organizations, used advertisement channels through mass media and partnered with religious organizations to recruit study participants. As the primary purpose of the forums was to educate community members about genetic ancestry testing; information on where participants were raised or the length of time at current residence was not collected [22]. All participants provided written informed consent. Participants also completed a 22-item questionnaire on demographics, knowledge, attitudes and beliefs about genetic testing. The study was approved by the Institutional Review Board at Howard University.

#### Measures

Age was denoted in *years*. Sex was categorized as *male* or *female*. Self-identified race/ethnicity or SIRE question was: How do you describe your ethnicity? Participants who selected *African American*, *East African*, *West African*, *Afro Caribbean*, *Central African* and *Mixed* were included in the study. Education was represented as total number of years of education completed. Marital Status question was: Which of the following describes your current situation? Response options included *Married*, *Widowed*, *Divorced*, *Separated*, *Never Married*, and *Other*. Employment Status included response options: *Not employed*, *Employed*, *Retired*, and *Other*. Occupation was an open response question [What is (was) your job?], and responses were then coded into the following categories: *Student/other*, *Unskilled*, *Skilled*, and *Professional*. Three independent coders developed the original categories. Any conflicts were discussed until agreement was reached between two or more coders. *Skilled* individuals held certifications, associate and bachelor level degrees and included individuals with experience in their respective discipline. *Professional* individuals held post-bachelor’s degrees (masters, doctorates) in their disciplines. *Unskilled* individuals were individuals that did not meet *skilled* and *professional* inclusion criteria. The total household income before taxes included responses ranging from *less than $10*,*000* to *at least $100*,*000*. Participants’ skin pigmentation was measured using a Derma-Spectrophotometer® (Cortex Technology) at two sites (forehead and upper inner arm), referenced in results as melanin index (MI) [4, 23]. At each site, three measurements were taken and then averaged to represent forehead and inner arm melanin index. Melanin index measurements range from 0–100% with higher values indicative of higher melanin content in the skin or darker skin and lower values reflecting less melanin content and lighter skin [24, 25].

DNA was genotyped for a previously validated set of 277 ancestry informative markers (AIMs) which was then used to estimate proportions of West African, Native American, and European genetic ancestry [26]. After genotyping quality control and filtering, ancestry admixture proportions were determined using a supervised model-based clustering algorithm in the STRUCTURE software [27, 28]. Mitochondrial DNA HVS-1 (primer set L16055 and H16410) was sequenced in both directions. The sequences were aligned and edited with the SeqMan program (DNASTAR, 1989–2003), resulting in a reading range of np 16072–16393. HVS-1 sequences are listed in S5 Table. Mutations in the HVI characteristic of specific mitochondrial haplogroups were determined using MITOMASTER (www.mitomap.org/foswiki/bin/view/mitomaster/webhome).

### Data analysis

Chi-square tests were performed to analyze sociodemographic differences of participants by city. Inner arm melanin index that represents constitutive pigmentation [4]—a polygenic trait that is comparatively unaffected by environmental factors—was used in correlation analyses to test differences between skin color and genetic ancestry. Multinomial logistic regression was performed to determine differences in socioeconomic status (including education) by skin color, and ancestry, pooled and stratified by study site, while controlling for age, ethnicity, marital, and employment status. Logistic regression analysis was used to examine the relationship between skin color and sex for having ever been married; age and site were included as covariates in the analysis. Data analyses were performed using SPSS version 25 and R statistical analysis software.

## Results

### Demographics

The study sample consists of 259 persons, 184 females (71.0%) and 75 males (29.0%), with a mean age of 49.8 years (range 18–80, SD = 15.64). As shown in Table 1, there were more African American participants in OH (90.4%), African/Caribbean participants in NY (11.5%) and mixed participants in OK (48.2%) than other cities. More individuals in DC (76.7%) reported they were employed than did respondents in OK (69.6%), OH (59.6%), and NY (44.9%). Additionally, most participants were educated professionals with an average household income between $25,000–99,000. A greater proportion of participants in NY were older (over than 65 years old), while those in OK, and OH were mostly between the ages of 45–64. DC participants were primarily between the ages of 18–44.

**Table 1:** Sociodemographic characteristics of Black participants (N = 259).

Variables	Sites				Total	
	OK	OH	NY	DC	n	%
	(*n* = 56)	(*n* = 52)	(*n* = 78)	(*n* = 73)		
SIRE*†						
African American	51.8%	90.4%	62.8%	82.2%	185	71.4
African or Caribbean	0.0%	1.9%	11.5%	8.2%	16	6.2
Mixed	48.2%	7.7%	25.6%	9.6%	58	22.4
Age (years)*						
18–44	34.5%	32.7%	29.9%	52.8%	97	37.5
45–64	47.3%	48.1%	35.1%	38.9%	106	40.9
≥65	18.2%	19.2%	35.1%	8.3%	53	20.5
**Sex**						
Male	35.7%	23.1%	25.6%	31.5%	75	29.0
Female	64.3%	76.9%	74.4%	68.5%	184	71.0
**Marital Status**						
Married	48.2%	40.4%	28.2%	31.9%	93	35.9
Widowed	10.7%	5.8%	10.3%	2.8%	19	7.3
Separated/Divorced	17.9%	26.9%	24.4%	26.4%	62	23.9
Never Married	23.2%	23.1%	34.6%	36.1%	78	30.1
Other	0.0%	3.8%	2.6%	2.8%	6	2.3
**Education**						
≤ High school	12.5%	7.7%	9.0%	2.7%	20	7.7
≤College degree	44.6%	63.5%	52.6%	54.8%	139	53.7
Graduate/professional	42.9%	28.8%	38.5%	42.5%	100	38.6
Employment Status*						
Not Employed	3.6%	3.8%	5.1%	5.5%	12	4.6
Employed	69.6%	59.6%	44.9%	76.7%	161	62.2
Retired	19.6%	26.9%	38.5%	13.7%	65	25.1
Other	7.1%	9.6%	11.5%	4.1%	21	8.1
**Occupation**						
Student/other	3.8%	6.4%	1.5%	1.5%	7	2.7
Unskilled	5.8%	2.1%	4.5%	3.1%	9	3.5
Skilled	28.8%	23.4%	31.3%	35.4%	70	27.0
Professional	61.5%	68.1%	62.7%	60.0%	145	56.0
**Household Income**						
Less than $10,000	9.4%	10.6%	5.3%	7.4%	18	6.9
$10,000–24,000	9.4%	10.6%	14.0%	10.3%	25	9.7
$25,000–49,000	39.6%	27.7%	31.6%	26.5%	70	27.0
$50,000–99,000	34.0%	40.4%	33.3%	32.4%	78	30.1
At least $100,000	7.5%	10.6%	15.8%	23.5%	34	13.1

Due to missing values, column percentages do not total 100%

Chi-square tests were performed for each demographic variable

*Significant level p < .05

**†**Self-identified race/ethnicity (SIRE)

### Skin color (melanin index) and social attainment

The pooled data on skin pigmentation variations across our study population is shown in Table 2. Participants in OH (46.99 ±1.06) had the highest average inner arm melanin index, followed by DC (46.88±0.96) and NY (45.54±0.96). Melanin index data were not collected for Oklahoma. In general, for men only regardless of skin color site measurement, men with higher education had darker skin (forehead: r = 0.345; inner arm measurement: r = 0.278). Furthermore, men with darker skin color (p = 0.047), and women with lighter skin (p = 0.021) were significantly more likely to be married. The interaction of sex, melanin index, and marriage was also significant, p = 0.04.

**Table 2:** Distribution of skin color (M index, inner arm) and ancestry (%) by SES* among Blacks from four US cities.

Characteristics	M index^†^	(SE)	p-value	%WAA^a^	(SE)	p-value	%EA^b^	(SE)	p-value	%NAA^c^	(SE)	p-value
**Occupation**			**0.03**			**0.02**			0.44			**0.00**
Unskilled	43.49	3.10		0.59	0.06		0.28	0.06		0.13	0.02	
Skilled	47.59	1.13		0.73	0.02		0.24	0.02		0.03	0.01	
Professional	46.44	0.76		0.72	0.02		0.25	0.01		0.03	0.01	
**Household Income**			0.19			0.32			0.84			0.48
Less than $10,000	49.01	2.36		0.72	0.04		0.24	0.04		0.04	0.02	
$10,000–24,000	45.44	1.80		0.72	0.04		0.23	0.03		0.05	0.01	
$25,000–49,000	46.21	1.19		0.72	0.02		0.24	0.02		0.04	0.01	
$50,000–99,000	47.28	1.03		0.72	0.02		0.24	0.02		0.04	0.01	
At least $100,000	45.58	1.58		0.70	0.03		0.25	0.03		0.05	0.01	
**Education**			**0.01**			*0*.*05*			0.47			0.35
≤ High school	43.88	2.08		0.67	0.04		0.26	0.03		0.07	0.01	
≤ College degree	46.18	0.78		0.70	0.02		0.24	0.01		0.06	0.01	
Graduate degree	47.20	0.90		0.71	0.02		0.24	0.02		0.05	0.01	
**Sex**			**0.01**			**0.03**			0.37			0.29
Male	45.96	1.05		0.68	0.02		0.27	0.02		0.05	0.01	
Female	46.61	0.66		0.71	0.01		0.25	0.01		0.04	0.01	
**Group**			**0.01**			**0.04**			0.21			0.14
OK	-	-		0.70	0.03		0.26	0.02		0.04	0.01	
OH	46.99	1.06		0.74	0.03		0.23	0.02		0.03	0.01	
NY	45.54	0.95		0.68	0.02		0.27	0.02		0.05	0.01	
DC	46.88	0.96		0.71	0.02		0.24	0.02		0.05	0.01	
**SIRE**^d^			**0.00**			**0.00**			**0.00**			**0.00**
AA	46.89	0.69		0.75	0.02		0.22	0.01		0.03	0.01	
Mixed	42.74	1.34		0.57	0.03		0.34	0.02		0.09	0.01	

^*^Socioeconomic status includes occupation, household income, and education

^**†**^Multinomial logistic regression analysis controlled for age, ethnicity, marital status, and employment status

^a^West African Ancestry

^b^European Ancestry

^c^Native American Ancestry

^d^Self-identified race/ethnicity (SIRE)

Additionally, individuals who were unskilled were significantly lighter with less West African ancestry, and more European and Native American ancestry than individuals with skilled and professional occupations. Darker skinned individuals with significantly higher West African ancestry reported attainment of a graduate degree, while lighter individuals reported completion of at least a high school diploma. Participants who self-reported as African American were darker in complexion than those who self-reported as mixed. African American participants also had higher West African ancestry, lower European and lower Native American ancestry than our mixed participants (Table 2). When we examined the differences across sites, education and occupation variables are nominally significant in NY and OH (S1 and S2 Tables) and ancestry varied across household income brackets in OK (S3 Table).

### Variations in skin color and ancestry

**Fig 1:**
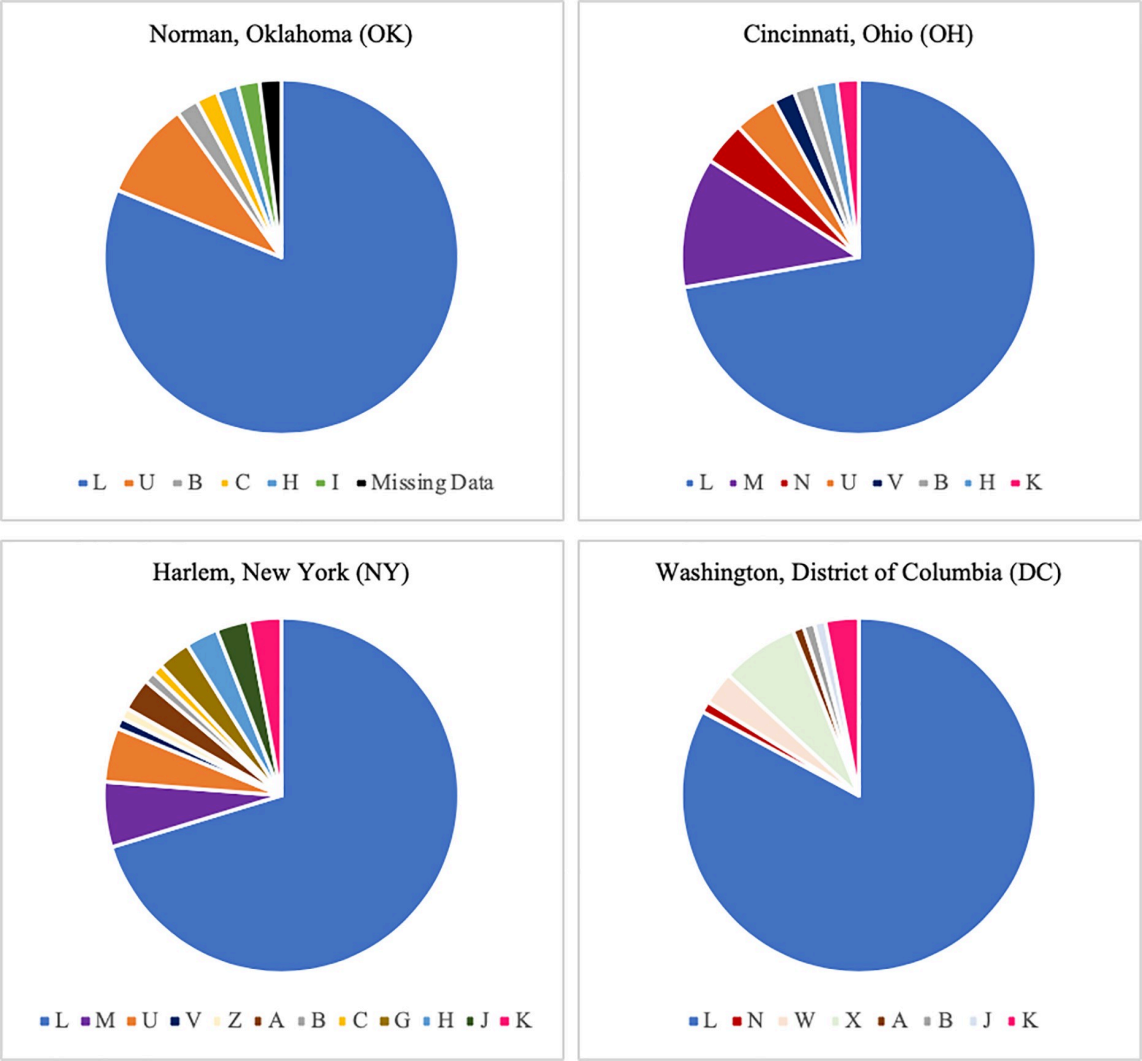
Mitochondrial DNA haplogroup pie charts with percentages. Mitochondrial DNA haplogroups. L—Africa; M, N—North East Africa/Middle East; A, B, C, D, X—Native American; A, B, C, D, F, G—Asia; H, I, J, N, U, K, V, J, Z, W—Europe.

S1 Fig shows the variation in skin color and West African ancestry across our study population in OH, NY, and DC. Overall, both men and women with higher West African ancestry had darker skin (men—forehead: r = 0.582; inner arm: r = 0.566 and women—forehead: r = 0.582; inner arm 0.532). Participants who identified as African American were significantly (p < .001) darker than participants who identified as being of “mixed” ancestry (Table 2). They also had significantly more West African ancestry and less European and Native American ancestry than our mixed participants. Additionally, regional differences related to skin pigmentation and genetic ancestry were evident. For example, participants in OH had significantly more West African ancestry than those in DC, NY, and OK. However, in NY, participants were more likely to be lighter with less West African ancestry, and more European and Native American ancestry. Fig 1 shows the percentages of mitochondrial macro haplogroups [5] by city.

**Fig 2:**
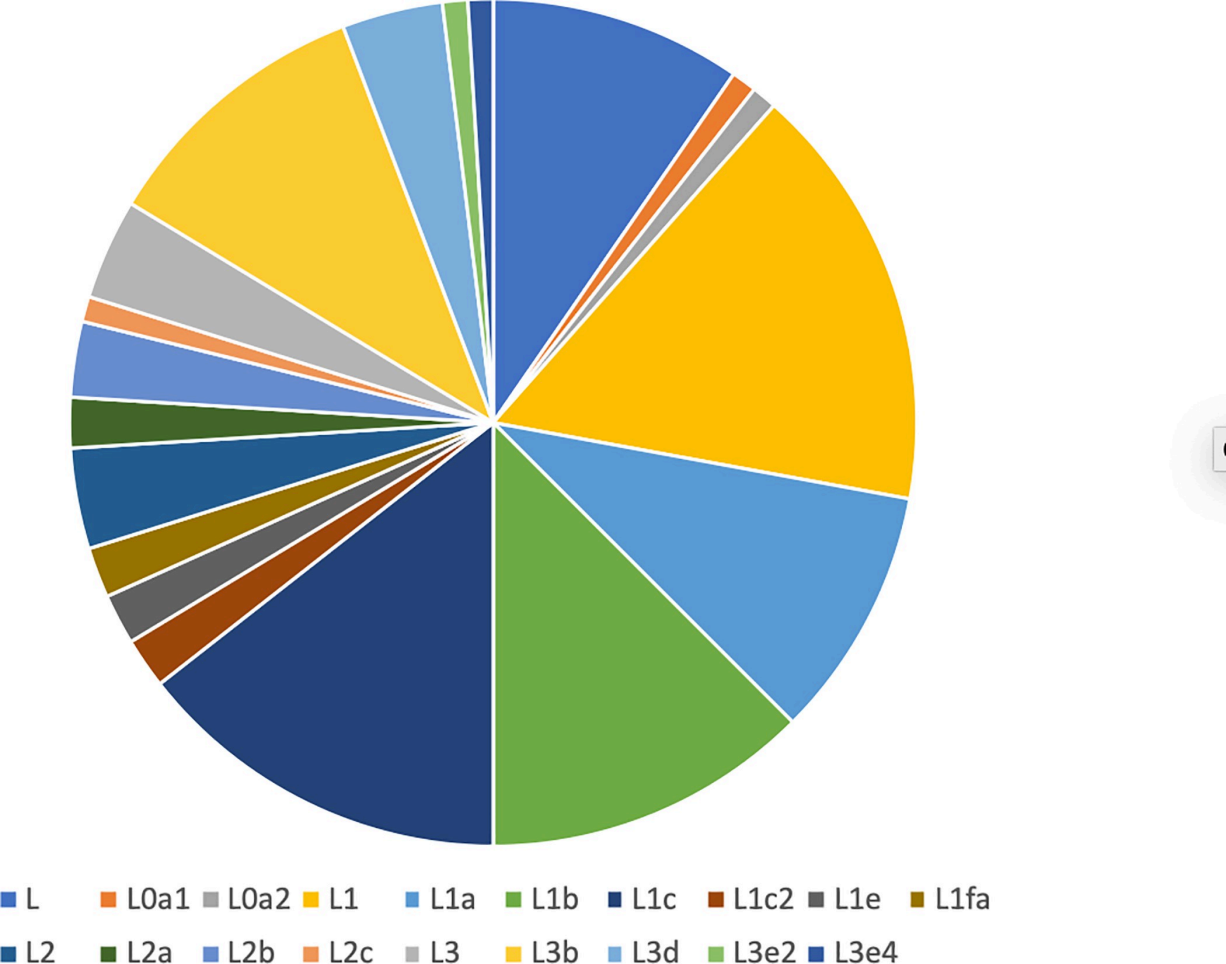
Diversity of mitochondrial DNA L-haplogroups among African Americans in the Four City Study.

Participants in OH had the highest percentage of the West African macro haplogroup L, while individuals in DC had the lowest percentage of the European haplogroups, but the highest percentage of Native American haplogroups. OK had the lowest percentage of Native American haplogroups and NY had the highest percentage of European haplogroups. Fig 2 reveals the diversity and number of L haplogroup lineages found among our study participants.

**Fig 3:**
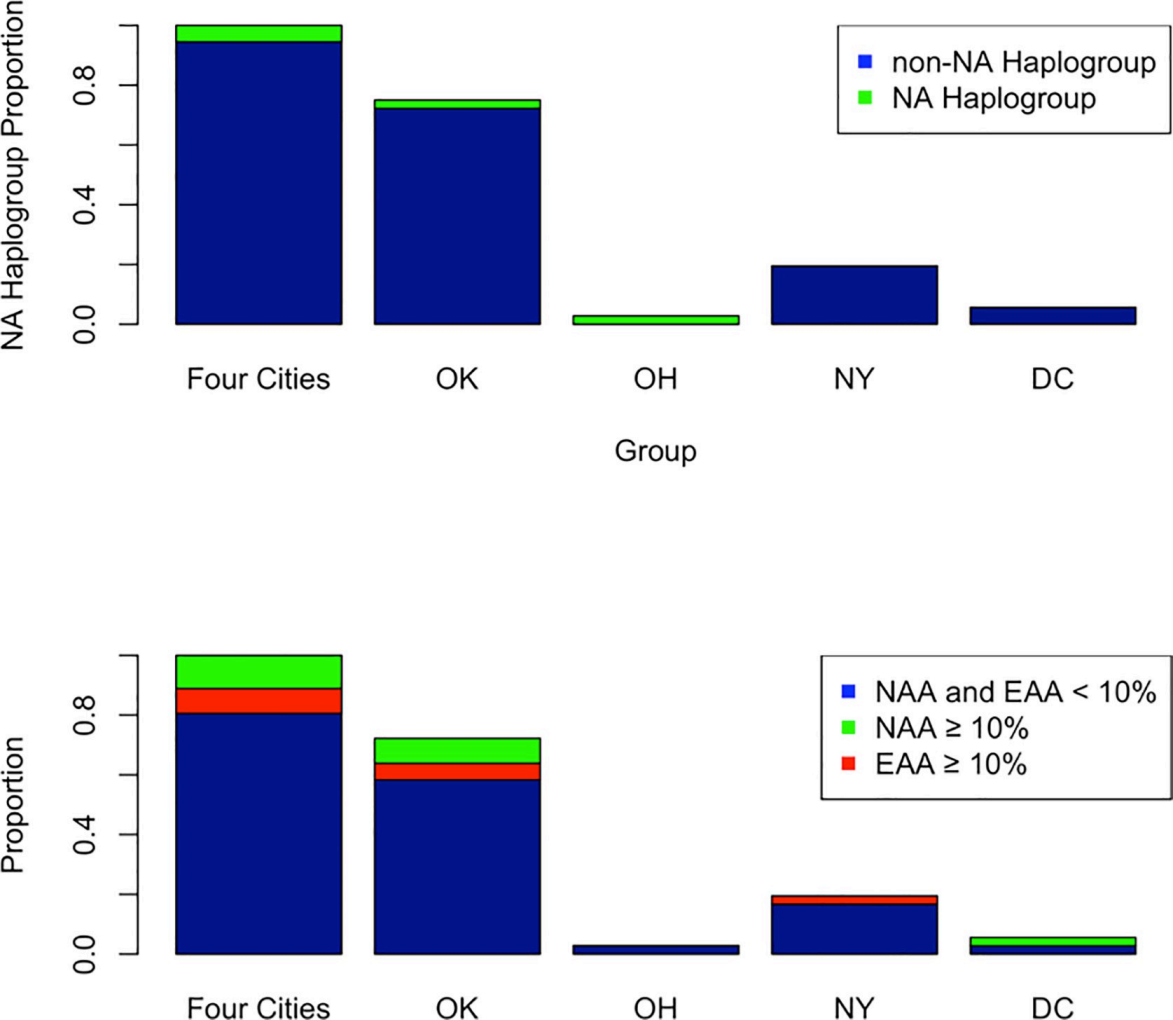
Self-identified race/ethnicity versus genetic evidence of Native American Ancestry (NAA) among mixed individuals. A. Individuals who identify as mixed show little Native American (NAA) maternal genetic ancestry as evidenced by mitochondrial DNA haplogroups B. Among mixed individuals that claim Native American Ancestry, few have greater than 10% global Native American or European American Genetic Ancestry (EAA).

Fig 3 shows the number of Native American haplotypes per city (upper) and mixed participants with greater than or equal to 10% Native American or European global ancestry per city (lower), per city, amongst individuals who claimed mixed ethnicity. While almost 50% of OK participants identified as mixed (Table 1), these participants had a nearly identical amount of Native American and European ancestry. No participant from OK or NY had greater than or equal 10% Native American ancestry. Overall, mixed participants across the four cities had more European than Native American ancestry (not shown).

## Discussion

Our study shows significant variation in skin color, genetic ancestry, and social attainment of Blacks across four cities in the United States. These findings both converge with and diverge from the existing literature on skin color. Scholars have reported that lighter skin is correlated with greater likelihood of being married [11], which we also found, and having better income and higher educational achievements [3]; but in our study, across some sites darker skinned participants were more educated and held skilled or professional jobs. Paradoxically, they also had the lowest household incomes compared to lighter skinned participants who were less educated and had lower level employment. Our finding regarding income is similar to other studies that have shown darker skinned individuals overall earned less than lighter skin counterparts with equivalent qualifications [3, 9, 10, 29].

Nearly 67% of our total sample were married at some point in their lives and only 33% of females in our study were never married. Men with darker skin and women with lighter skin were more likely to be married. Our general findings align with those from Udry and colleagues [11] that lighter skinned women and darker skinned men were more advantageous for mate selection prospects than both groups’ respective counterparts. However, their findings were limited to participants from DC, while our study sample included data from NY and OH. Our study confirms what has been previously found about the sexual dimorphism [30–32] of skin color in Blacks, and further strengthens the evidence base on the differences between men and women regarding the impact of skin color on marriage prospects. These findings may also be explained by the idolization of Eurocentric beauty standards in the United States that stems from slavery, the emancipation proclamation, Jim Crow and the New Jim Crow [33]. According to sociologists Keith and Herring [10], Whites placed a superior economic value on slaves of mixed ancestry and treated those slaves “better” than those who were predominantly of West African ancestry or darker skin. After the emancipation proclamation of 1863 [34], the idea stated by Hunter [3] that “if you’re light you’re alright”, appears to continue to be perpetuated in both Black and White communities across the United States.

Skin pigmentation is the most conspicuous trait known to humans. Our findings suggest skin pigmentation differs by geography and ancestry. This supports previous research that shows regional variations in the ancestry of U.S. Blacks. For instance, research using mitochondrial DNA (which is maternally inherited) has found that African Americans and Caribbean Blacks residing along the east coast have lower Native American and European ancestry [35, 36]. While this previous finding held true for our participants in DC, in NY, participants had the least West African ancestry and more European and Native American ancestry. These differences within our sample and across studies could be attributed to migration history, social norms, mate-selection, and local history [21]. For example, there is a large and growing population of people from the Dominican Republic [37] and Puerto Rico living in New York, and both groups have been shown to have more European ancestry [38, 39]. It is plausible that intermarriage may have altered the gene flow over time, thereby increasing the proportion of European ancestry found among Blacks in NY.

There may be several reasons why participants in OH had significantly more West African ancestry than those in DC and were darker than participants in NY. Social norms and local history may provide some explanation. During the era of the Underground Railroad, fugitive enslaved Africans traveled through or resided in OH with Native Americans and/or developed their own siloed communities [40]. The absence of gene flow (admixture) between Native Americans and European Americans with the fugitive enslaved Africans due to opposition against ending slavery may have protected the dilution of West African ancestry.

On the contrary, gene flow between Europeans and enslaved Blacks in OK, not Native Americans, may explain the proportion of ancestry for participants from that region. For some Blacks in OK, integration with Native Americans was embraced after the Emancipation Proclamation of 1863 [15, 41]. The Cherokee, for example, kept their enslaved Blacks until 1866, and the former enslaved, also known as *Freedmen*, were granted full tribal citizenship. However, the creation of the Dawes Commission or the General Allotment Act of 1887, forcing all Cherokee citizens to carry a *Certificate of Degree of Indian Blood* (i.e., confirmation of Native American blood), resulted in the exclusion of *Freedmen* from voting, participating in tribal councils, and the dismissal of benefits or rights ascribed to tribal citizens [15]. The actions against the *Freedmen* then continue to inform how their descendants are treated today. For example, for Black individuals living in Tahlequah, OK, the hope to use DNA testing to confirm their Native American citizenry was dashed [41]. While some Blacks could socially trace their lineage to the *Trail of Tears*, their DNA test results did not confirm their Native American blood line. With the growing popularity of DNA testing informing one’s understanding of cumulative life experiences [42] and health risk [43, 44], the question arises regarding its utility in confirming or refuting genealogy relative to entitlement of rights or benefits espoused to specific groups [45–47].

The traditional paradigm of using race as a proxy for ancestral background in biomedical research is slowly shifting given the heterogeneity that exists in U.S. populations. This is especially the case for research on African Americans and Hispanic/Latino Americans. In these recently admixed populations, continental genetic contribution or biogeographic ancestry may be estimated using ancestry informative markers (AIMs). AIMs are genetic markers, typically SNPs, which are found across the human genome and have large allele frequency differences between continental groups such as Western Europeans and West Africans and are powerful for estimating biogeographic ancestry [5]. Genetic ancestry for each individual is estimated by comparing individual’s AIM genotypes to that of a reference panel consist of samples from continental ancestral populations using a statistical probability modeling approach. Continental ancestral genetic contributions estimated using AIMs illustrate the fluidity and variation of genetic ancestry within traditional U.S. “racial” groups. Levels of Euorpean admixture in African American populations vary across the U.S. African Americans from the southern states tend to have lower levels of European admixture, while much higher estimates of European ancestry is observed in the Pacific North West [48, 49]. This geographic distribution of genetic ancestry should be interpreted in terms of well-known historical and demographic events that have played an important role in African American history [50].

Despite the differences found among Blacks in this study and others, race remains an prominent variable in social science and biomedical health research [4, 5, 20, 21, 35]. Genetic ancestry is not always correlated with SIRE (self-identified race/ethnicity), which is largely based on skin color. The relationship between skin color, ancestry, and disease is complex and is influenced by cultural norms, history, and behavior [21, 51, 52]. These sociopolitical and personal factors influence disparities in health outcomes among so-called racial and ethnic groups [21, 53–56]. The correlation between race and disease is largely due to the interaction between an individual’s skin color with society (racism) [21]. For example, the association between genetic ancestry, skin color, and blood pressure may be attributed to sociocultural factors related to race and racism [53, 54, 57]. A study on glucocorticoid receptor signaling and stress in African American and European American men with prostate cancer indicated cumulative stressful life events (psychosocial stressors) may play a role in aggressive prostate cancer phenotype and mortality in African American men [56]. Psychosocial stressors (perceived discrimination) based on race have been shown to be significantly associated with detrimental health outcomes for African American, and Caribbean American adults [55]. Considering the differences identified among Blacks across the four cities in our study, it may be useful to also consider genetic ancestry and local historical/social context in research. Doing so may provide a more robust understanding of the relationship between our genes, the environment (social and physical), and disease.

### Strengths and limitations

This study expands our knowledge of the complexity of race, ancestry, and social attainment across four cities in the United States. Our findings support the heterogeneity of the Black population in the U.S. that could be influenced by regional differences and local history. This is the first study, to our knowledge, to examine the intersectionality among race, ancestry and social attainment in four cities across the US. While a notable strength, due to the timing of data collection, we were unable to collect skin color data from our OK participants. The modest sample size, a population primarily of middle-aged respondents, lack of extensive genealogical data on the participants, and the cross-sectional nature of our methodology also limits generalizability and causal inferences of results. It is important to also note DNA testing was provided at no cost to participants, which may have introduced some respondent biases (e.g., desirability bias).

## Conclusion

Historically, skin pigmentation has been used to stratify populations into racially classified social groups that inform opportunity structures and social attainment via racism and systemic discrimination. Through this study, we have demonstrated that the U.S. is in fact heterogeneous in genetic and social status. Thus, Black U.S. populations should not be viewed as a singular entity, but as individual groups with their own local histories and genetic backgrounds.

## Supporting information

S1 Fig Relationship between skin color (M index, inner arm) and West African genetic ancestry (%) in black participants from Cincinnati, Ohio, Harlem, New York, and Washington, DC. (TIFF)

S1 Table Distribution of skin color (M index, inner arm) and ancestry (%) by SES* among blacks in Cincinnati, OH. *Socioeconomic status includes occupation, household income, and education ^†^Multinomial logistic regression analysis controlled for age, ethnicity, marital status, and employment status ^a^West African Ancestry ^b^European Ancestry ^c^Native American Ancestry.
(DOCX)

S2 Table Distribution of skin color (M index, inner arm) and ancestry (%) by SES* among blacks in Harlem, NY. *Socioeconomic status includes occupation, household income, and education ^†^Multinomial logistic regression analysis controlled for age, ethnicity, marital status, and employment status ^a^West African Ancestry ^b^European Ancestry ^c^Native American Ancestry.
(DOCX)

S3 Table Distribution of ancestry (%) by SES* among blacks in Norman, Oklahoma. *Socioeconomic status includes occupation, household income, and education ^†^Multinomial logistic regression analysis controlled for age, ethnicity, marital status, and employment status ^a^West African Ancestry ^b^European Ancestry ^c^Native American Ancestry.
(DOCX)

S4 Table Distribution of skin color (M index, inner arm) and ancestry (%) by SES* among blacks in Washington, DC. *Socioeconomic status includes occupation, household income, and education ^†^Multinomial logistic regression analysis controlled for age, ethnicity, marital status, and employment status ^a^West African Ancestry ^b^European Ancestry ^c^Native American Ancestry.
(DOCX)

S5 Table mtDNA HVS-1 sequence variants among study subjects. (XLSX)

S1 File Dataset for “genetic ancestry, skin color and social attainment: The four cities study”. (SAV)
