# Supplementary figures and images for: Genetic ancestry, skin color and social attainment: The four cities study
Source: PLoS One. 2020 Aug 19;15(8):e0237041. doi: 10.1371/journal.pone.0237041 (PMC7446776; doi:10.1371/journal.pone.0237041)

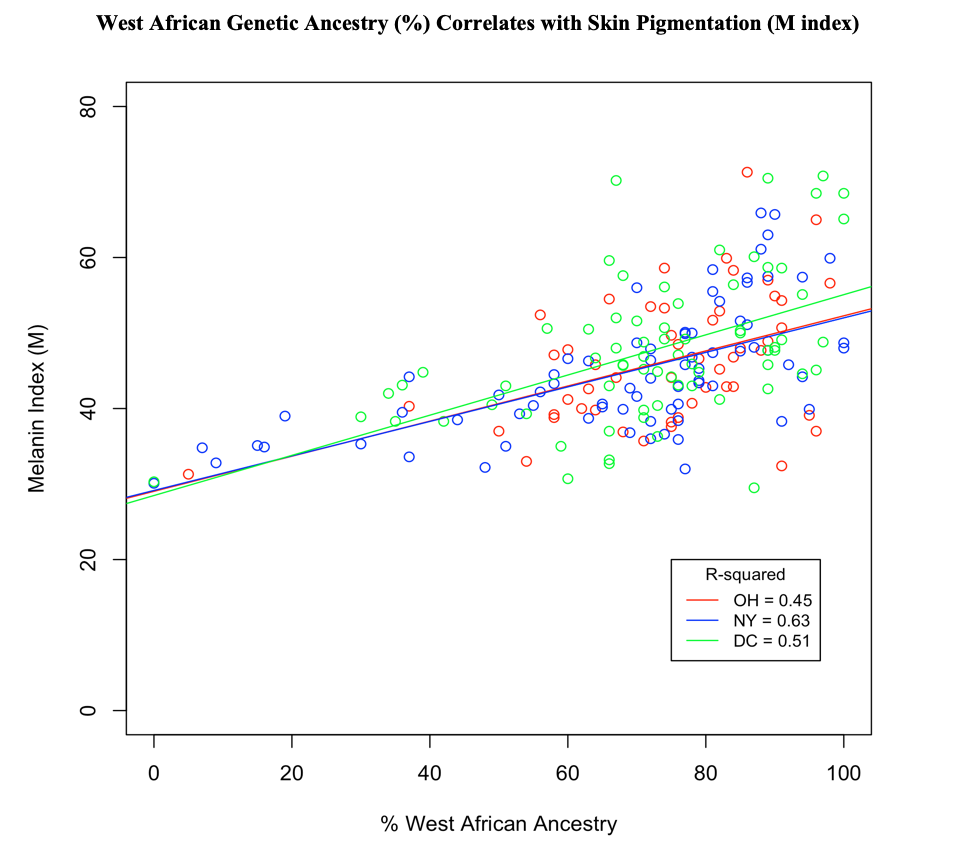

Supplement: S1 Fig — (TIFF) [file pone.0237041.s001.tiff]
