# Supplementary material for: Genetic ancestry, skin color and social attainment: The four cities study
Source: PLoS One. 2020 Aug 19;15(8):e0237041. doi: 10.1371/journal.pone.0237041 (PMC7446776; doi:10.1371/journal.pone.0237041)
Supplement: S1 Table — *Socioeconomic status includes occupation, household income, and education †Multinomial logistic regression analysis controlled for age, ethnicity, marital status, and employment status aWest African Ancestry bEuropean Ancestry cNative American Ancestry. (DOCX) [file pone.0237041.s002.docx]

**S1 Table.** Distribution of skin color (M index, inner arm) and ancestry (%) by SES^*^ among Blacks in Cincinnati, OH

| **Characteristics** | M index**^†^** | (SE) | p-value | %WAA^a^ | (SE) | | p-value | | %EA^b^ | | (SE) | p-value | %NAA^c^ | (SE) | p-value |
| --- | --- | --- | --- | --- | --- | --- | --- | --- | --- | --- | --- | --- | --- | --- | --- |
| **Occupation** |  |  | **0.00** |  | |  | | 0.17 | |  |  | 0.25 |  |  | 0.54 |
| Unskilled | 61.60 | 5.60 |  | 0.90 | | 0.14 | |  | | 0.04 | 0.12 |  | 0.06 | 0.04 |  |
| Skilled | 50.34 | 1.98 |  | 0.82 | | 0.05 | |  | | 0.14 | 0.04 |  | 0.04 | 0.01 |  |
| Professional | 45.88 | 1.17 |  | 0.72 | | 0.03 | |  | | 0.23 | 0.02 |  | 0.05 | 0.01 |  |
| **Household Income** |  |  | 0.18 |  | |  | | 0.43 | |  |  | 0.56 |  |  | 0.93 |
| Less than $10,000 | 46.95 | 4.42 |  | 0.82 | | 0.08 | |  | | 0.15 | 0.07 |  | 0.03 | 0.02 |  |
| $10,000-24,000 | 44.52 | 3.19 |  | 0.74 | | 0.06 | |  | | 0.22 | 0.05 |  | 0.04 | 0.02 |  |
| $25,000-49,000 | 44.51 | 2.19 |  | 0.70 | | 0.04 | |  | | 0.25 | 0.04 |  | 0.05 | 0.01 |  |
| $50,000-99,000 | 49.91 | 1.78 |  | 0.77 | | 0.03 | |  | | 0.18 | 0.03 |  | 0.05 | 0.01 |  |
| At least $100,000 | 46.79 | 3.45 |  | 0.81 | | 0.06 | |  | | 0.14 | 0.05 |  | 0.05 | 0.02 |  |
| **Education** |  |  | *0.07* |  | |  | | 0.74 | |  |  | 0.93 |  |  | 0.64 |
| ≤ High school | 41.21 | 3.87 |  | 0.83 | | 0.08 | |  | | 0.15 | 0.07 |  | 0.02 | 0.02 |  |
| ≤ College degree | 47.17 | 1.36 |  | 0.75 | | 0.03 | |  | | 0.21 | 0.02 |  | 0.04 | 0.01 |  |
| Graduate degree | 47.83 | 1.66 |  | 0.71 | | 0.04 | |  | | 0.22 | 0.03 |  | 0.04 | 0.01 |  |
| **Gender** |  |  | *0.06* |  | |  | | 0.61 | |  |  | 0.84 |  |  | 0.52 |
| Male | 46.17 | 2.02 |  | 0.73 | | 0.04 | |  | | 0.22 | 0.04 |  | 0.05 | 0.01 |  |
| Female | 47.29 | 1.15 |  | 0.74 | | 0.02 | |  | | 0.21 | 0.02 |  | 0.05 | 0.01 |  |

**^*^**Socioeconomic status includes occupation, household income, and education

**^†^**Multinomial logistic regression analysis controlled for age, ethnicity, marital status, and employment status

^a^West African Ancestry

^b^European Ancestry

^c^Native American Ancestry
