# Supplementary material for: Genetic ancestry, skin color and social attainment: The four cities study
Source: PLoS One. 2020 Aug 19;15(8):e0237041. doi: 10.1371/journal.pone.0237041 (PMC7446776; doi:10.1371/journal.pone.0237041)
Supplement: S2 Table — *Socioeconomic status includes occupation, household income, and education †Multinomial logistic regression analysis controlled for age, ethnicity, marital status, and employment status aWest African Ancestry bEuropean Ancestry cNative American Ancestry. (DOCX) [file pone.0237041.s003.docx]

**S2 Table.** Distribution of skin color (M index, inner arm) and ancestry (%) by SES^*^ among Blacks in Harlem, NY

| **Characteristics** | M index**^†^** | (SE) | p-value | %WAA^a^ | (SE) | | p-value | | %EA^b^ | | (SE) | p-value | %NAA^c^ | (SE) | p-value |
| --- | --- | --- | --- | --- | --- | --- | --- | --- | --- | --- | --- | --- | --- | --- | --- |
| **Occupation** |  |  | **0.04** |  | |  | | **0.001** | |  |  | 0.29 |  |  | **0.001** |
| Unskilled | 36.80 | 0.78 |  | 0.32 | | 0.10 | |  | | 0.41 | 0.10 |  | 0.27 | 0.14 |  |
| Skilled | 45.79 | 1.88 |  | 0.66 | | 0.06 | |  | | 0.31 | 0.06 |  | 0.03 | 0.01 |  |
| Professional | 45.24 | 1.30 |  | 0.71 | | 0.03 | |  | | 0.25 | 0.02 |  | 0.04 | 0.01 |  |
| **Household Income** |  |  | 0.58 |  | |  | | 0.19 | |  |  | 0.84 |  |  | 0.18 |
| Less than $10,000 | 45.47 | 3.97 |  | 0.66 | | 0.08 | |  | | 0.29 | 0.07 |  | 0.05 | 0.01 |  |
| $10,000-24,000 | 47.30 | 4.12 |  | 0.63 | | 0.10 | |  | | 0.24 | 0.08 |  | 0.13 | 0.08 |  |
| $25,000-49,000 | 44.32 | 1.95 |  | 0.66 | | 0.06 | |  | | 0.27 | 0.04 |  | 0.07 | 0.04 |  |
| $50,000-99,000 | 43.79 | 2.29 |  | 0.66 | | 0.06 | |  | | 0.26 | 0.05 |  | 0.08 | 0.03 |  |
| At least $100,000 | 44.33 | 2.07 |  | 0.70 | | 0.08 | |  | | 0.25 | 0.08 |  | 0.05 | 0.01 |  |
| **Education** |  |  | *0.09* |  | |  | | **0.018** | |  |  | 0.41 |  |  | 0.14 |
| ≤ High school | 41.21 | 2.58 |  | 0.50 | | 0.10 | |  | | 0.38 | 0.09 |  | 0.12 | 0.08 |  |
| ≤ College degree | 45.37 | 1.61 |  | 0.67 | | 0.04 | |  | | 0.26 | 0.04 |  | 0.07 | 0.02 |  |
| Graduate degree | 45.02 | 1.45 |  | 0.72 | | 0.03 | |  | | 0.23 | 0.03 |  | 0.05 | 0.01 |  |
| **Gender** |  |  | *0.07* |  | |  | | **0.024** | |  |  | 0.35 |  |  | 0.12 |
| Male | 42.26 | 1.80 |  | 0.60 | | 0.06 | |  | | 0.32 | 0.04 |  | 0.08 | 0.03 |  |
| Female | 45.85 | 1.21 |  | 0.69 | | 0.03 | |  | | 0.26 | 0.03 |  | 0.05 | 0.01 |  |

**^*^**Socioeconomic status includes occupation, household income, and education

**^†^**Multinomial logistic regression analysis controlled for age, ethnicity, marital status, and employment status

^a^West African Ancestry

^b^European Ancestry

^c^Native American Ancestry
