# Supplementary material for: Genetic ancestry, skin color and social attainment: The four cities study
Source: PLoS One. 2020 Aug 19;15(8):e0237041. doi: 10.1371/journal.pone.0237041 (PMC7446776; doi:10.1371/journal.pone.0237041)
Supplement: S3 Table — *Socioeconomic status includes occupation, household income, and education †Multinomial logistic regression analysis controlled for age, ethnicity, marital status, and employment status aWest African Ancestry bEuropean Ancestry cNative American Ancestry. (DOCX) [file pone.0237041.s004.docx]

**S3 Table.** Distribution of ancestry (%) by SES^*^ among Blacks in Norman, Oklahoma

| **Characteristics** | M index**^†^** | (SE) | p-value | %WAA^a^ | (SE) | | p-value | | %EA^b^ | | (SE) | p-value | %NAA^c^ | (SE) | p-value |
| --- | --- | --- | --- | --- | --- | --- | --- | --- | --- | --- | --- | --- | --- | --- | --- |
| **Occupation** |  |  |  |  | |  | | 0.33 | |  |  | 0.14 |  |  | 0.32 |
| Unskilled | N/A | -- | -- | 0.74 | | 0.12 | |  | | 0.24 | 0.12 |  | 0.02 | 0.03 |  |
| Skilled | N/A | -- | -- | 0.73 | | 0.05 | |  | | 0.21 | 0.05 |  | 0.06 | 0.01 |  |
| Professional | N/A | -- | -- | 0.66 | | 0.04 | |  | | 0.28 | 0.04 |  | 0.06 | 0.01 |  |
| **Household Income** |  |  |  |  | |  | | **0.02** | |  |  | **0.01** |  |  | **0.02** |
| Less than $10,000 | N/A | -- | -- | 0.65 | | 0.08 | |  | | 0.24 | 0.08 |  | 0.11 | 0.02 |  |
| $10,000-24,000 | N/A | -- | -- | 0.83 | | 0.10 | |  | | 0.14 | 0.09 |  | 0.03 | 0.02 |  |
| $25,000-49,000 | N/A | -- | -- | 0.74 | | 0.05 | |  | | 0.22 | 0.05 |  | 0.04 | 0.01 |  |
| $50,000-99,000 | N/A | -- | -- | 0.67 | | 0.05 | |  | | 0.28 | 0.05 |  | 0.05 | 0.01 |  |
| At least $100,000 | N/A | -- | -- | 0.52 | | 0.09 | |  | | 0.45 | 0.08 |  | 0.03 | 0.02 |  |
| **Education** |  |  |  |  | |  | | **0.04** | |  |  | **0.03** |  |  | 0.40 |
| ≤ High school | N/A | -- | -- | 0.57 | | 0.07 | |  | | 0.39 | 0.07 |  | 0.04 | 0.02 |  |
| ≤ College degree | N/A | -- | -- | 0.68 | | 0.04 | |  | | 0.28 | 0.04 |  | 0.04 | 0.01 |  |
| Graduate degree | N/A | -- | -- | 0.75 | | 0.05 | |  | | 0.20 | 0.04 |  | 0.05 | 0.01 |  |
| **Gender** |  |  |  |  | |  | | **0.02** | |  |  | **0.01** |  |  | 0.30 |
| Male | N/A | -- | -- | 0.68 | | 0.04 | |  | | 0.26 | 0.04 |  | 0.06 | 0.01 |  |
| Female | N/A | -- | -- | 0.70 | | 0.03 | |  | | 0.25 | 0.03 |  | 0.05 | 0.01 |  |

**^*^**Socioeconomic status includes occupation, household income, and education

**^†^**Multinomial logistic regression analysis controlled for age, ethnicity, marital status, and employment status

^a^West African Ancestry

^b^European Ancestry

^c^Native American Ancestry
