# Supplementary material for: Genetic ancestry, skin color and social attainment: The four cities study
Source: PLoS One. 2020 Aug 19;15(8):e0237041. doi: 10.1371/journal.pone.0237041 (PMC7446776; doi:10.1371/journal.pone.0237041)
Supplement: S4 Table — *Socioeconomic status includes occupation, household income, and education †Multinomial logistic regression analysis controlled for age, ethnicity, marital status, and employment status aWest African Ancestry bEuropean Ancestry cNative American Ancestry. (DOCX) [file pone.0237041.s005.docx]

| **Characteristics** | M index**^†^** | (SE) | p-value | %WAA^a^ | (SE) | | p-value | | %EA^b^ | | (SE) | p-value | %NAA^c^ | (SE) | p-value |
| --- | --- | --- | --- | --- | --- | --- | --- | --- | --- | --- | --- | --- | --- | --- | --- |
| **Occupation** |  |  | 0.71 |  | |  | | 0.53 | |  |  | 0.79 |  |  | 0.57 |
| Unskilled | 48.10 | - |  | 0.90 | | - | |  | | 0.10 | - |  | 0.00 | - |  |
| Skilled | 47.30 | 1.80 |  | 0.71 | | 0.04 | |  | | 0.25 | 0.03 |  | 0.04 | 0.01 |  |
| Professional | 48.14 | 1.37 |  | 0.71 | | 0.03 | |  | | 0.25 | 0.02 |  | 0.04 | 0.01 |  |
| **Household Income** |  |  | 0.53 |  | |  | | 0.68 | |  |  | 0.91 |  |  | 0.37 |
| Less than $10,000 | 54.00 | 5.10 |  | 0.77 | | 0.04 | |  | | 0.21 | 0.04 |  | 0.02 | 0.00 |  |
| $10,000-24,000 | 44.61 | 1.24 |  | 0.67 | | 0.09 | |  | | 0.29 | 0.08 |  | 0.04 | 0.02 |  |
| $25,000-49,000 | 47.68 | 2.12 |  | 0.69 | | 0.06 | |  | | 0.26 | 0.04 |  | 0.05 | 0.02 |  |
| $50,000-99,000 | 47.62 | 1.65 |  | 0.72 | | 0.03 | |  | | 0.25 | 0.03 |  | 0.03 | 0.01 |  |
| At least $100,000 | 45.62 | 2.51 |  | 0.74 | | 0.03 | |  | | 0.23 | 0.03 |  | 0.03 | 0.01 |  |
| **Education** |  |  | 0.20 |  | |  | | 0.40 | |  |  | 0.76 |  |  | 0.34 |
| ≤ High school | 56.25 | 6.06 |  | 0.82 | | 0.06 | |  | | 0.16 | 0.05 |  | 0.02 | 0.01 |  |
| ≤ College degree | 47.01 | 1.29 |  | 0.74 | | 0.03 | |  | | 0.22 | 0.02 |  | 0.04 | 0.01 |  |
| Graduate degree | 47.27 | 1.55 |  | 0.73 | | 0.03 | |  | | 0.24 | 0.03 |  | 0.03 | 0.01 |  |
| **Gender** |  |  | 0.39 |  | |  | | 0.41 | |  |  | 0.73 |  |  | 0.25 |
| Male | 49.50 | 2.30 |  | 0.72 | | 0.23 | |  | | 0.23 | 0.04 |  | 0.05 | 0.02 |  |
| Female | 46.68 | 1.02 |  | 0.72 | | 0.19 | |  | | 0.24 | 0.02 |  | 0.04 | 0.01 |  |

**S4 Table.** Distribution of skin color (M index, inner arm) and ancestry (%) by SES^*^ among Blacks in Washington, DC

**^*^**Socioeconomic status includes occupation, household income, and education

**^†^**Multinomial logistic regression analysis controlled for age, ethnicity, marital status, and employment status

^a^West African Ancestry

^b^European Ancestry

^c^Native American Ancestry
